# Socioeconomic inequities of COVID-19 mortality in vulnerable Comunas of the City of Buenos Aires

**DOI:** 10.1038/s41598-023-40911-1

**Published:** 2023-08-22

**Authors:** Agustina M. Marconi, Carlos Castillo Salgado, Elena Beatriz Sarrouf, Rafael Jose Zamora, Alejandra Maria Irurzun, Nazrul Islam

**Affiliations:** 1 University Health Services, University of Wisconsin Madison, 333 East Campus Mall, Madison, WI 53715 USA; 2 Department of Epidemiology at, Johns Hopkins University, 615 N Wolfe St, Baltimore, MD 21205 USA; 3 Direction of Epidemiology, Province of Tucuman, Virgen de la Merced 196, San Miguel de Tucumán, Tucumán Argentina; 4 Medicus, Larrea 877, Buenos Aires, Argentina; 5 Sub-Secretary of Primary, Ambulatory and Community Care of the CABA, Monasterio 480, 1283 Buenos Aires, Argentina; 6 Faculty of Medicine, University of Southampton, Southampton, UK

**Keywords:** Public health, Quality of life

## Abstract

During the COVID-19 pandemic, the gap in health inequities was exposed and increased, showing how different vulnerable groups were affected. Our aim was to examine the correlation between an area-based health inequity index and mortality due to COVID-19 in people 60 years old or above in the City of Buenos Aires in 2020. We developed a Health Inequity Composite Index (HICI), including six core indicators. Each indicator value per Comuna was first standardized to a Z-score. All six Z-scores were summed into a final composite Z-score to rank the Comunas from lowest to highest social inequities. Comunas from the northern part of the city had lower inequities whereas those in the south had higher levels of inequities. COVID-19 age-standardized mortality rate in people 60 years or above was higher in the Comunas from the south and lower in those from the north. Finally, we found a strong positive correlation (Rho = 0.83, p < 0.0001 CI95% = 0.65–0.99) between HICI and age-standardized mortality rates from COVID-19 in people 60 years or above. Our finding of a strong correlation between the levels of health inequity and mortality calls for a concerted effort in narrowing or eliminating existing inequities.

## Introduction

There is substantiated evidence that health inequities have a spatial footprint, often following the geographical patterns of inequity in the social, economic, and physical environmental conditions in which people are born, grow, live, work and age^1,2^. These gaps had profound impacts on health. In England, for example, people living in the poorest neighborhoods die on average seven years earlier than people living in the richest neighborhoods and the average difference in disability-free life expectancy is even greater, 17 years^3^. In Malmö, Sweden, this difference is up to eight years depending on which part of the city people live in^4^. Even within disadvantaged groups in a city, the causes of inequities in health may differ by sex and by age, as studies of women in Madrid and adolescents in Barcelona have shown, indicating the complexity of the issue^5,6^. In Latin America, one of the most unequal regions in the world, there is also evidence of the impact of health inequities in urban areas^7,8^. 

From the beginning of the COVID-19 pandemic, the inequities in health outcomes were exposed and widened, showing how different vulnerable groups and areas were severely affected^9^. Amongst those socially disadvantaged clusters, elderly people have been disproportionately affected by COVID-19, with adults aged 60 or over accounting for over 95% of deaths in Europe^10^.

Urban areas showed structural inequities both in terms of number of cases and deaths due to COVID-19^11^. This was particularly true in Latin America where densely populated cities with concentrated poverty were disproportionately affected^12^. Numerous studies have examined the relationship between social disadvantage and COVID-19 mortality rates in different Latin American countries. For instance, research conducted in Brazil^13^, Chile^14^, Colombia^15^, and Mexico^16^ consistently reported positive associations between indicators of social disadvantage and COVID-19 mortality rates. These findings suggest that individuals from socioeconomically disadvantaged backgrounds are at a higher risk of mortality due to the virus. In contrast, no significant association between poverty and mortality was observed in the metropolitan area of Lima Peru^17^. These divergent findings highlight the complex interplay between social determinants of health and COVID-19 outcomes, emphasizing the importance of examining these relationships within specific localities.

The Autonomous City of Buenos Aires (CABA) is an autonomous Argentinean jurisdiction with one of the best socio-demographics and health indicators in the country. In 2020, data showed CABA had the higher human development index by jurisdiction (0.885) and the lower unsatisfied basic needs (UBN) of 7%^18^. Despite these apparent positive indicators, large cities usually have differences within their territories (neighborhoods) in their health, socioeconomic and demographic indicators^19^. CABA also presents important and visible socioeconomic inequities when assessing different areas of the city specially north/south, or when looking at its administrative division called Comunas^7^. The CABA has an estimated population in 2020 of 3,075,646 inhabitants, with a slightly higher proportion of females. Recent demographic trend shows a higher proportion of older adults and women in the population^20^. Data from 2020 shows a negative natural growth of −2.4 with gross birth and death rates of 9.1 and 11.6 per thousand inhabitants, respectively^20^. This data reflects a stationary population pyramid, with 16.3% of the population aged 65 years old or above, compared to an 11.5% of population 65 or above for the entire country.^21,22^. Quantifying the association between health inequity and health outcomes would help inform resource prioritization for the local health policy. It will also improve our understanding on the importance of collection, availability, analysis, and potential impact on social and health indicators to examine health inequities, especially in the context of large metropolitan areas around the world.

The aim of the study is to examine the correlation between the area-based health inequity index and mortality due to COVID-19 in the population 60 years or above in 2020, in the City of Buenos Aires. We hypothesize a positive correlation between the level of health inequity and COVID-19 mortality in the population 60 years or above.

For this analysis, we used the following definition for the concept inequity: referred to unfair, avoidable differences arising from poor governance, corruption or cultural exclusion. Health inequities are differences in health status or on the distribution of health resources between different population groups, arising from the social conditions in which people are born, grow, live, work and age. Health inequities are systematic differences in health outcomes^23^.

## Results

**Figure 1:**
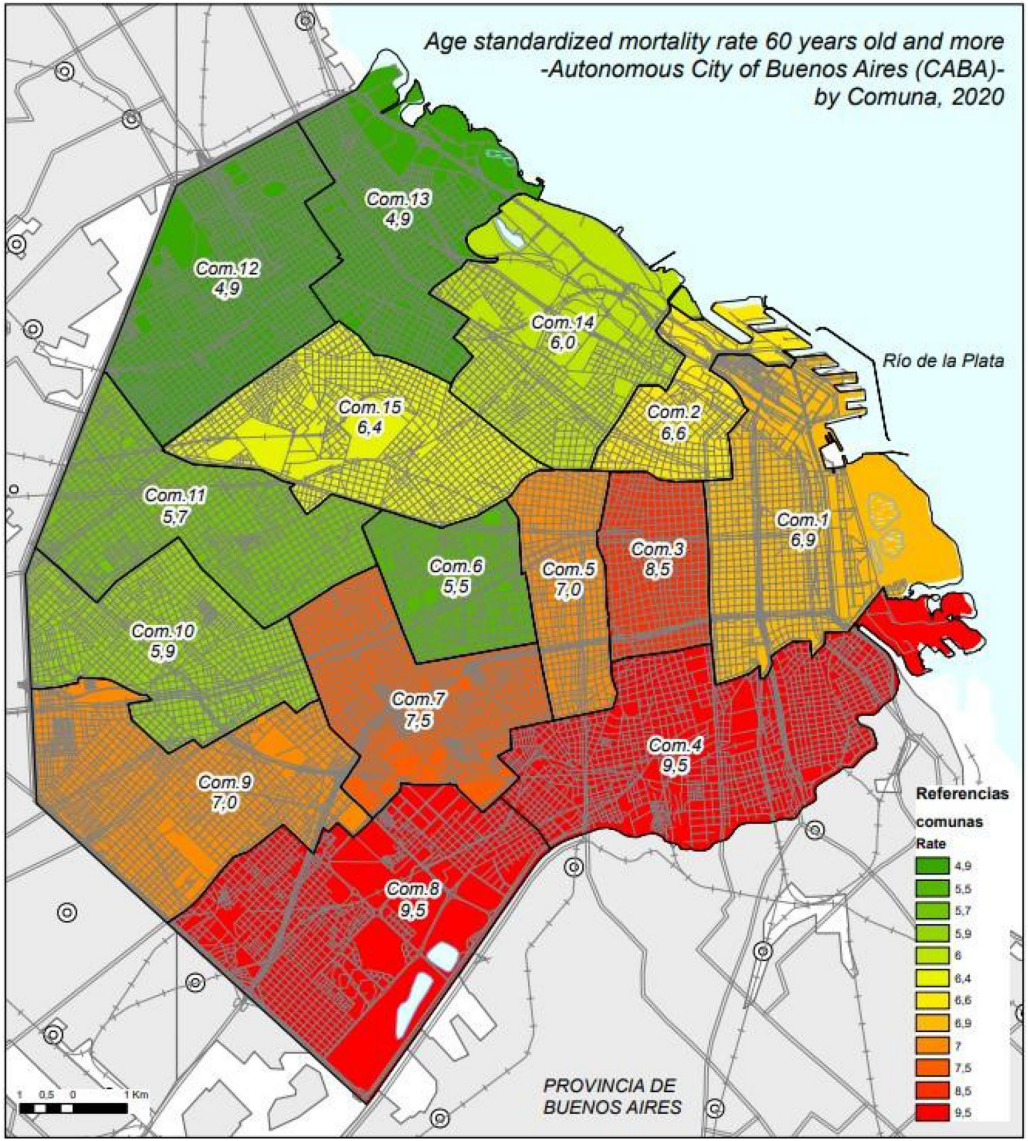
Age standardized mortality rate 60 years or above (%) by Comuna. City of Buenos Aires (CABA) Argentina, 2020. Source: This figure generated by the authors with data provided by the National Statistics and Health Information Direction, National Health Minister and data available in the Direction of Statistics and Census of the City of Buenos Aires using the program ArcGIS. https://www.arcgis.com/index.html.

Table 1 shows that Comunas in the south part of the city (4 and 8) had a higher percentage in five of the six chosen indicators: residents of 25 years or above with high school degree or less, adolescent birth rate, percentage of households with income lower than total living expenses, percentage of population with public health system only, and percentage of households without sewage connection. Age-standardized mortality rate was also high in these Comunas in the south. In contrast, Comunas in the north part of the city (13, 14 and 15) had lower percentages of the selected indicators. Comuna 13 had the lowest age-standardized mortality rate. COVID-19 age-standardized mortality rate in 60 years or above was higher in Comunas from the south (9.5% for both Comunas 4 and 8) and lower in the northern Comunas (Fig. 1 and Supplementary Fig. 1).

**Table 1:** Descriptive statistics per Comuna for six chosen indicator. City of Buenos Aires (CABA), Argentina. 2020.

Comuna	Demographic indicators			Socioeconomic indicators	Health indicators		Environmental indicators	
	Age-standardized mortality rate in people 60 years or above (per 1000)	Residents aged ≥ 25 years with high school degree or less 2021 (%)	Adolescent birth rate (live birth to female aged 15–19 per 1000 females aged 10–19 years) 2019–2021	Households with income lower to total living expenses 2021 (%)	Age-standardized mortality rate 2020 (per 1000)	Population with public health system only 2021 (%)	Households without sewage connection 2021 (%)	Households without sewage connection 2021; log-transformed (+ 1)
1	6.9	25.4	15.4	35.7	9.7	29.2	2.1	0.49
2	6.6	9.1	2.1	22.0	10.2	6.1	0.3	0.11
3	8.5	19.7	10.4	33.1	11.5	19.8	0.4	0.15
4	9.5	35.0	17.6	42.0	12.1	36.9	3.5	0.65
5	7.0	13.9	5.7	16.9	10.5	9.6	0.4	0.15
6	5.5	12.1	3.8	17.2	9.6	8.9	0.2	0.08
7	7.5	21.2	12.6	31.5	10.6	24.4	1.1	0.32
8	9.5	46.5	17.1	59.2	11.3	48.8	4.6	0.75
9	7.0	29.5	10.0	30.3	11.1	25.9	1.1	0.32
10	5.9	24.1	7.2	25.9	10.4	18.1	0.5	0.18
11	5.7	16.4	4.4	16.0	10.5	11.1	0.4	0.15
12	4.9	13.0	4.1	15.8	9.6	4.2	0.5	0.18
13	4.9	5.0	2.6	13.2	9.3	5.7	0.2	0.08
14	6.0	6.1	2.2	17.2	9.7	5.0	0.2	0.08
15	6.4	16.5	6.2	10.8	10.7	13.1	0.8	0.26
Mean (x̄)	6.8	19.6	8.1	25.8	10.4	17.8	1.1	0.26
Standard deviation (s)	1.5	11.3	5.4	13.1	0.8	13.1	1.3	0.21
Coefficient of variation	0.2	0.6	0.7	0.5	0.1	0.7	1.2	0.81

**Figure 2:**
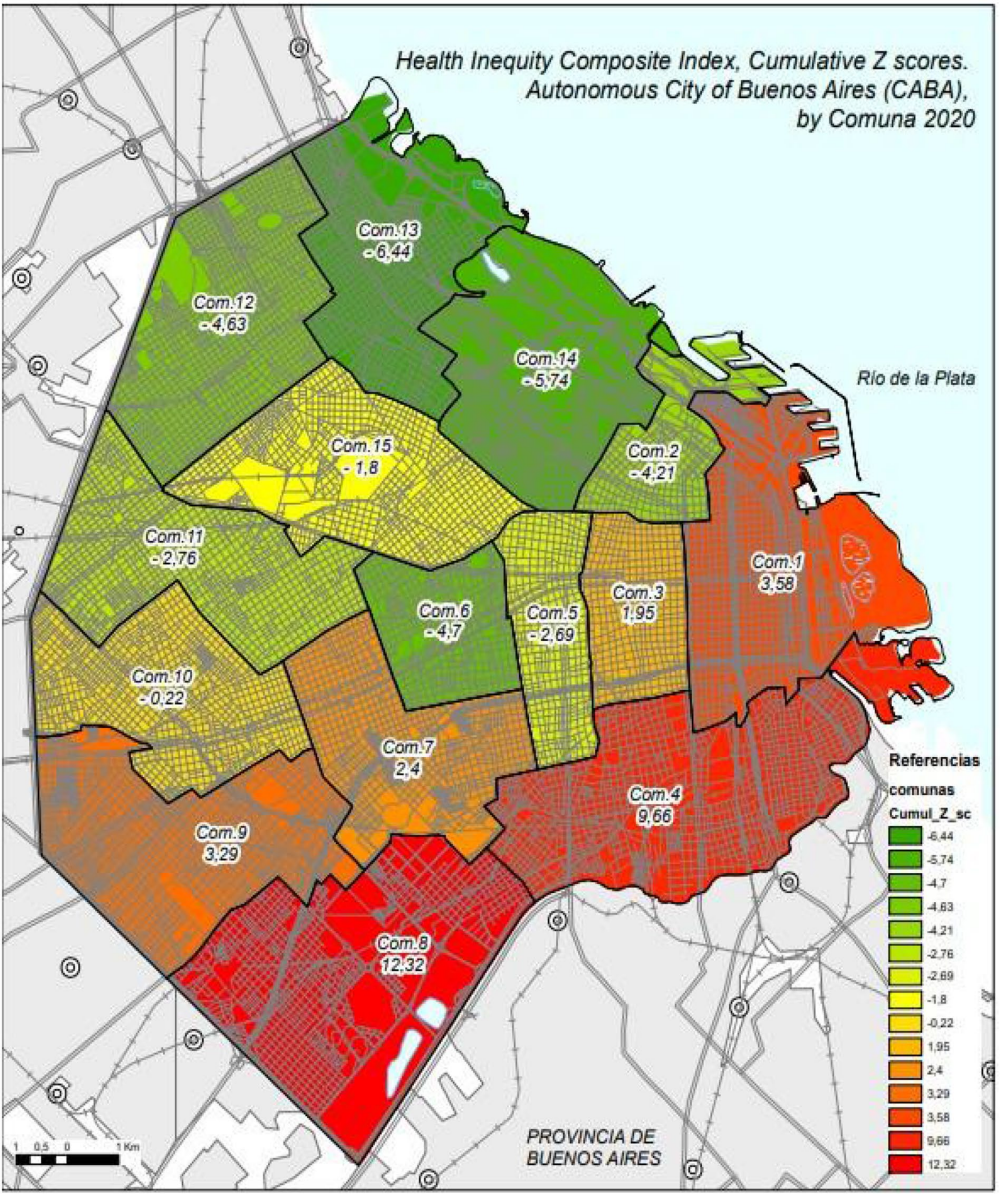
Health inequity composite index (HICI), by Comuna. City of Buenos Buenos Aires (CABA) Argentina, 2020. Source: This figure generated by the authors with data provided by the National Statistics and Health Information Direction, National Health Minister and data available in the Direction of Statistics and Census of the City of Buenos Aires using the program ArcGIS. https://www.arcgis.com/index.html\.

Table 2 shows that each HICI indicator had a positive directionality (i.e., a higher value indicates a greater health inequity). HICI was lower in Comunas from the northern part of the city and higher in those in the south. The least unequal Comunas were the Comuna 2 (Z-score: −4.2), 6 (−4.7), 13 (−6.4) and 14 (−5.7). The Comunas with greater inequities were 4 (9.7), and 8 (12.3), both in the south part of the city (Fig. 2). The Z-score distance of 18.8 represents the maximum width of inequity between the least unequal Comuna. The Supplementary Fig. 2 shows the relationship between indicators (Z score) per Comuna. It shows that the HICI indicators were positively correlated and concentrated in southern Comunas showing a pattern of syndemics.

**Table 2:** Z-score transformation of indicator values by Comuna. City of Buenos Aires (CABA), Argentina. 2020.

Comuna	Demographic indicators		Socioeconomic indicators	Health indicators	Environment indicators			
	Residents aged ≥ 25 years with high school degree or less 2021 (%)	Adolescent birth rate (live birth to female aged 15–19 per 1000 females aged 10–19 years) 2019–2021	Households with income lower to total living expenses 2021 (%)	Age-standardized mortality rate in 2020 (per 1000)	Population with public health system only 2021 (%)	Households without sewage connection 2021 (%); log-transformed (+ 1)		Cumulative Z score
1	0.5	1.3	0.8	−1.0	0.9	1.1		3.58
2	−0.9	−1.1	−0.3	−0.3	−0.9	−0.7		−4.21
3	0.0	0.4	0.6	1.4	0.2	−0.5		1.95
4	1.4	1.7	1.2	2.0	1.5	1.8		9.66
5	−0.5	−0.4	−0.7	0.1	−0.6	−0.5		−2.69
6	−0.7	−0.8	−0.7	−1.1	−0.7	−0.9		−4.70
7	0.1	0.8	0.4	0.2	0.5	0.3		2.40
8	2.4	1.7	2.6	1.1	2.4	2.3		12.32
9	0.9	0.3	0.3	0.8	0.6	0.3		3.29
10	0.4	−0.2	0.0	−0.1	0.0	−0.4		−0.22
11	−0.3	−0.7	−0.7	0.0	−0.5	−0.5		−2.76
12	−0.6	−0.7	−0.8	−1.1	−1.0	−0.4		−4.63
**13**	−1.3	−1.0	−1.0	−1.4	−0.9	−0.9		−6.44
14	−1.2	−1.1	−0.7	−1.0	−1.0	−0.9		−5.74
15	−0.3	−0.3	−1.1	0.4	−0.4	0.0		−1.80

Z-Score: (xi − x̄)/s.

**Figure 3:**
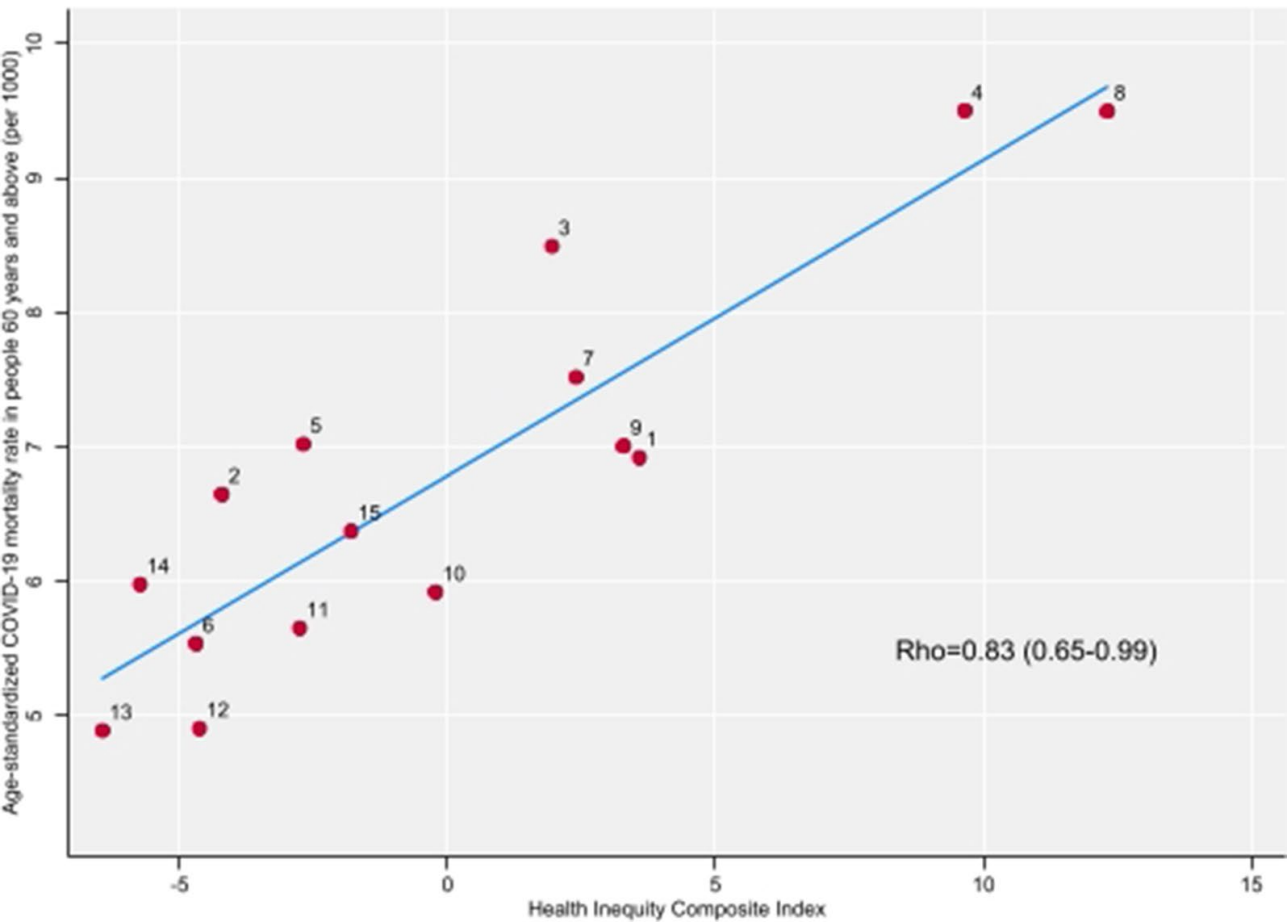
Scatterplot of age-standardized mortality rate from COVID-19 in people 60 years or above in 2020 and the HICI, for each Comuna. City of Buenos Aires, 2020. Source: Generated by the authors with data provided by the National Statistics and Health Information Direction, National Health Minister and data available in the Direction of Statistics and Census of the City of Buenos Aires.

Figure 3 shows a very high positive correlation between age-standardized mortality rates from COVID-19 in people 60 years or above and the HICI (Rho = 0.83; p < 0.0001 CI95% = 0.65–0.99). Comunas 4 and 8 in the south were in the top right of the scatter plot (high levels of HICI and COVID-19 age-standardized mortality) while Comunas 12 and 13 in the north in the bottom left of the plot (low levels of HICI and COVID-19 mortality).

Table 3 shows the absolute and relative difference in the indicator values of the respective Comuna and those in the Comuna with the lowest cumulative HICI score (Comuna 13). Compared with the least deprived Comuna (Comuna 13), the most deprived one (Comuna 8) had a 41.5% (9.3 times, on a ratio scale) higher percentage of residents aged ≥ 25 years with high school degree or less, 14.5% (6.7 times) higher adolescent birth rate, 46% (4.5 times)higher percentage of households with income lower than total living expenses, a 2% (1.2 times) higher age-standardized mortality rate, a 43.1% (8.6 times) higher percentage of population with public health system only, and 4.4% (23 times) higher percentage of households without sewage connection system.

**Table 3:** Measures of c inequity per Comuna relative to the Comuna with lowest deprivation. City of Buenos Aires, Argentina 2020.

Comuna	Residents aged ≥ 25 years with high school degree or less 2021 (%)		Adolescent birth rate (live birth to female aged 15–19 per 1000 females aged 10- 19 years) 2019–2021		Households with income below total living expenses 2021 (%)		Age-standardized mortality rate in 2020 per 1000 inhabitants		Population with public health system only 2021(%)		Households without sewage connection 2021 (%); log-transformed (+ 1)	
	Absolute difference	Ratio	Absolute difference (‰)	Ratio	Absolute difference	Ratio	Absolute difference (‰)	Ratio	Absolute difference	Ratio	Absolute difference	Ratio
13 (reference)	0	1	0	1	0	1	0	1	0	1	0	1
1	20.4	5.1	12.8	6.0	22.5	2.7	0.3	1.0	23.5	5.1	1.9	10.5
2	4.1	1.8	−0.5	0.8	8.8	1.7	0.9	1.1	0.4	1.1	0.1	1.5
3	14.7	3.9	7.8	4.0	19.9	2.5	2.2	1.2	14.1	3.5	0.2	2.0
4	30.0	7.0	15.0	6.8	28.8	3.2	2.7	1.3	31.2	6.5	3.3	17.5
5	8.9	2.8	3.1	2.2	3.7	1.3	1.2	1.1	3.9	1.7	0.2	2.0
6	7.1	2.4	1.3	1.5	4.0	1.3	0.3	1.0	3.2	1.6	0.0	1.0
7	16.2	4.2	10.0	4.9	18.3	2.4	1.3	1.1	18.7	4.3	0.9	5.5
8	41.5	9.3	14.5	6.7	46.0	4.5	2.0	1.2	43.1	8.6	4.4	23.0
9	24.5	5.9	7.4	3.9	17.1	2.3	1.8	1.2	20.2	4.5	0.9	5.5
10	19.1	4.8	4.6	2.8	12.7	2.0	1.0	1.1	12.4	3.2	0.3	2.5
11	11.4	3.3	1.8	1.7	2.8	1.2	1.1	1.1	5.4	1.9	0.2	2.0
12	8.0	2.6	1.5	1.6	2.6	1.2	0.2	1.0	−1.5	0.7	0.3	2.5
14	1.1	1.2	−0.4	0.9	4.0	1.3	0.3	1.0	−0.7	0.9	0.0	1.0
15	11.5	3.3	3.7	2.4	−2.4	0.8	1.4	1.1	7.4	2.3	0.6	4.0

Comuna 13 was used as the reference since it had the lowest cumulative HICI Z-score.

## Discussion

In this analysis, we adapted a published methodology on health inequities^24^ by including different health, educational and environmental indicators to create a summary HICI for each Comuna, the minimal geographical unit with systematic statistical data in the CABA^24^. We then assessed its correlation with mortality due to COVID-19 in the population 60 years or above.

Our results revealed significant differences in health indicators and mortality rates across different Comunas of Buenos Aires. Comunas in the south had higher percentages in five out of six selected indicators, including educational attainment, adolescent birth rate, income inequality, public health system coverage, and lack of sewage connection. In contrast, the northern Comunas generally had lower percentages of the analyzed indicators. Our findings are consistent with the historical analysis that highlights how Comunas 4 and 8 alternates in the podium of the worst health, and socioeconomic outcomes over time^25,26^. Comunas in the south of the city also had a higher COVID-19 age age-standardized mortality rate in older adults exhibiting a very strong correlation between the inequities and COVID-19 mortality. Similar findings were reported in another study using a different methodological approach, including spatial clustering of population density and basic unmet needs, and different geographical units found higher mortality in population 60 years or above specially during the first wave^27^. Nevertheless, our research used updated data from a larger geographical unit, the “Comunas” as the city of Buenos Aires has a very robust history of data in the level of Comuna that could expand the analysis to pre and post pandemic era^20^. Moreover, we created a relatively simple index with publicly available data in most of the provinces in Argentina, which makes our methodology easily replicable in other jurisdictions.

Evidence on health inequities within cities has been globally documented across many countries, regardless of the level of economic development and health system organization^28–30^. It is known that places where people live within a city can shape individual and population health and create social inequities^31^. Globally, COVID-19 pandemic widened health inequities in those inhabitants more socially disadvantaged showing a phenomenon known as “syndemic pandemic” of higher mortality and morbidity rates among these groups^32,33^.

The impact of COVID-19 pandemic in Latin America had reached the levels of a humanitarian crisis, amplifying the effects of structural socioeconomic inequities in the region, where vulnerable population had been specially affected^34,35^. Moreover, even though mortality in older adults was higher during the pandemic, survey data from the region showed deepening inequities in healthcare^36^. More than 50 percent of respondents in lower income households reported problems with their food supply and they were also more likely to have difficulty purchasing medicines -more than twice the rates in high-income households^37^.

Like many other large cities in Latin America, the CABA does not escape the fragmentation of its urban structure, a consequence of the historical processes including migratory waves, economic crisis, to name a few^38^. Those geographical areas are territories with differential social realities that are usually masked under larger jurisdictional analysis^39^.

Our study has several strengths. First, we assessed the association between area-level inequity and COVID-19 mortality using robust and recent data which adds to the existing body of literature on widening inequities during the pandemic. Second, we developed a simple measure of a composite index of inequity using publicly available data. This could be replicated relatively easily elsewhere in the world. Third, the findings from our study show that our index is highly correlated with mortality indicating a high degree of validity of our measure. Additionally, the utilization of the novel Health Inequity Composite Index (HICI) enhances our understanding of health inequities experienced by disadvantage population and highlights the importance of addressing local contextual factors in combating the inequities.

This study has several limitations. This is a cross- sectional study, so no directionality of associations can be assessed. Within the unit of analysis, the Comunas, there are still hidden inequities. Although the designation slums, settlements, transitory housing nucleus makes the most vulnerable populations appear as small spots on the map of the City, these are formal denominations, typical of special cadasters that makes invisible a significant number of inhabitants who despite the fact living within a communal division have a greater risk of negative health outcomes than the average population of the city and their own Comuna^39^. As an example, Comuna 1 has impoverished settlements such as “Villas 31, 31 bis and the Barrio San Martin”, all three in the Retiro neighborhood, one of the wealthiest areas in the city, and the “Rodrigo Bueno” settlement in the neighborhood of Costanera Sur, also a very rich area.

Our findings highlight the importance of identification and analysis of the gaps in the living conditions of cities, requires the disaggregation of health outcomes at the neighborhood or the minimum administrative subunit of the city's inhabitants. They also underscore the need to identify areas that are disproportionately affected with a view to allocating healthcare resources proportionately through the lens of health equity and justice. Moving forward, it is imperative to implement integrated strategies and policies, such as a health-in-all-policies approach, that can effectively bridge these gaps and promote equitable health outcomes.

## Methods

### Data

Data for this study included health, income, education, and structural indicators, openly available on the Direction of Statistics and Census of the City of Buenos Aires’ web page^25^. We also used 2020 data of mortality from the National Statistics and Health Information Direction National Health Minister, and COVID-19 Mortality in people 60 years or above^40^.

### Geographical units

The City of Buenos Aires is administratively divided in 15 Comunas. Each Comuna has several neighborhoods within its boundaries. The Comunas are the minimal geographical unit with systematic data through the years and it is the unit chosen for this analysis^41^.

Several statistics reports nucleate the Comunas in four larger regions^42^. South Region: Comunas 4, 7, and 8; Center East Region: Comunas 1, 3, 5, 6, and 15; West Region: Comunas 9, 10, and 11; North Region: Comunas 2, 12, 13, and 14.

### Selection of core indicators

Core indicators are summary measures of specific domains that help monitor and assess social and health related trends over time. We used demographic, socioeconomic, health and environmental indicators across “Comunas” to characterize social and health inequities. The indicators were chosen considering direct or indirect relevance to health outcomes and systematic data availability. Due to the pandemic, there are almost no updated indicators in 2020, so we chose data from 2019 and 2021. Ideal indicators had sufficient variability to reflect the distribution of the risk factor in the population as well as to discriminate between areas of high and low inequities.

#### Population over 25 years old with incomplete high school degree or less

The educational level attained has traditionally been selected as a socioeconomic indicator because it is a predictor not only of the quality and condition of employment but also of income and the social and cultural context. It is also considered a structural indicator that remains stable with economic fluctuations. It is widely known that adults with higher education levels live healthier and longer lives when compared to less educated peers^43^. We used 2021 data^44^.

#### Adolescent birth rate (live birth to females aged 15–19 per 1000 females aged 10–19 years)

Much has been discussed about the association between pregnancy and poverty. This is certainly an indicator that behaves differently according to socioeconomic levels. In adolescent pregnancy, the reproductive behavior of adolescent mothers and the socioeconomic conditions in which they live determine the sexual practices, endorsed, and reinforced by the context^45^. The characteristics make this indicator eligible. We used 2021 data^42^.

#### Percentage of households with income lower to total living expenses

This is an indicator of deprivation, it is also an indicator sensitive to the economic situation of a country and specifically refers to the ability of a household to meet the cost of food that satisfies their food needs and some non-food goods and services such as clothing, transportation, education, health, housing, etc^46^. We used 2021 data^42^.

#### Households without sewage connection

The sewage system is the urban means of elimination of excreta. Disposal through sewerage is considered a basic need. The lack of connection to this system is an indicator of deprivation that is associated with structural poverty, and it is directly related to negative health outcomes including mortality^47^. We used 2021 data^48^.

#### Age-standardized mortality rate

This indicator was selected to account for the risk of dying that the inhabitants of each Comuna have regardless of the influence of its population structure. The age-standardization of death was done following Pan American Health Organization (PAHO) Guidance^49^. We used 2020 data of mortality from the National Statistics and Health Information Direction, National Health Minister, and Argentinian population 2010 for age standardization^40,50^.

#### Population with public health system only

Argentina’s health system is divided into three subsectors: public, private, and social security that coexist and overlap. The private subsector is paid out of pocket voluntarily. The social security sector is financed through regular fixed contributions from employees and employers in the formal working force. Finally, people living in Argentina, regardless of their Nationality or if they are covered under any of the other subsectors, can access the public subsector^51^. Due to this, the public health system has long waiting lists. Most of the population using exclusively the public subsector cannot afford private health insurance and are not covered through the social security sector assured by a formal job^52^. Like many indicators, it has socioeconomic characteristics, since it is directly related with employment status and salary. We used 2021 data^42^.

### Health inequity composite index (HICI)

We developed a Health Inequity Composite Index including six indicators to assess the overall magnitude and the relative Health inequities between the Comunas. The indicator value for each of the fifteen Comunas was first standardized to a Z-score: Z=$({\mathrm{x}}_{\mathrm{i}}- \overline{\mathrm{x} })/$s, where x_i = Communas-specific values, x = overall mean of the values, s = standard deviation. Indicators for which a high value reflects a higher health or lower social inequity were multiplied by + 1, whereas indicators for which a high value reflects a lower health or higher social inequity were multiplied by − 1. All six Z-scores for each Comuna were summed into a final composite Z-score to rank the 15 Comunas from lowest to highest health inequity.

We calculated the mean, standard deviation, and coefficient of variation of each indicator. One of the indicators, “percentage of households without sewage connection”, was log-transformed due to a coefficient of variation equal or greater than 100%. Other indicators were approximately normally distributed. Using mean and standard deviation, we obtained each Z-score and the cumulative Z-score. A higher HICI index indicates a higher level of socioeconomic deprivation. We used the Comuna with lower cumulative Z as the reference to compare the HICI within Comunas. Measures of absolute health inequity were calculated as the difference between indicator values in each Comuna and the reference Comuna (with the lowest cumulative HICI Z-score) to characterize the overall burden of inequity for each indicator. Measures of relative health inequity were also calculated as the ratio of each indictor values and the reference Comuna to compare inequities across health outcomes that use different scales. Using these two measures of inequity can help track both the reduction in inequity between groups and the overall elimination of the inequity altogether.

### COVID-19 mortality in people 60 years or above

The target population mortality in people 60 years or above as evidence shows older adults were at higher risk of mortality due to COVID-19 during pandemic months of 2020^1^. We used age-standardized mortality rate in 60 years or above (%).

To assess the correlation between mortality from COVID-19 and the HICI we calculated the specific mortality rates (COVID-19) in people 60 years or above by Comuna. We used official secondary data sources from statistical mandatory death certificates with a cause of death labeled as COVID-19 (U07 of the ICD-10), and 2020 population projections^53^. We age-standardized the mortality rate using Argentina 2010 Census population^40^.

### Ethical considerations

The study did not require evaluation by the ethics committee because it used secondary, publicly available data with no identifiable information^54^.

### Supplementary Information

Supplementary Figures.

## Data Availability

Links for publicly available datasets for the variables used to develop the HICI: https://www.estadisticaciudad.gob.ar/eyc/?page_id=35782\. Links for mortality data: https://www.estadisticaciudad.gob.ar/eyc/?cat=292. Links to Vital statistics: https://www.argentina.gob.ar/salud/deis/datos. The datasets related to mortality due to COVID-19 used and/or analyzed during the current study available from the corresponding author on reasonable request.
