# Supplementary material for: Socioeconomic inequities of COVID-19 mortality in vulnerable Comunas of the City of Buenos Aires
Source: Sci Rep. 2023 Aug 22;13:13642. doi: 10.1038/s41598-023-40911-1 (PMC10444792; doi:10.1038/s41598-023-40911-1)
Supplement: Supplementary file 1 — Supplementary Figures. [file 41598_2023_40911_MOESM1_ESM.docx]

**Supplementary Materials**

**Socioeconomic inequities of COVID-19 mortality in vulnerable Comunas of the City of Buenos Aires**

Agustina M. Marconi*, M.D., M.P.H^1^, Carlos Castillo Salgado, M.D., M.P.H., DrPH^2^, Elena Beatriz Sarrouf M.D., M.Sc.^3^, Rafael Jose Zamora M.D., M.B.A^4^, Alejandra Maria Irurzun B.Sc., M.P.H^5^, Nazrul Islam, M.B.B.S, MSc, M.P.H, Ph.D.^6^

1. University Health Services. University of Wisconsin Madison. 333 East Campus Mall Madison, 53715. Wisconsin. USA. Email: [agustina.marconi@wisc.edu](mailto:agustina.marconi@wisc.edu)
2. Department of Epidemiology at Johns Hopkins University. 615 N Wolfe St, Baltimore, MD 21205. Email: [ccastil3@jhu.edu](mailto:ccastil3@jhu.edu)
3. Direction of Epidemiology, province of Tucuman. Virgen de la Merced 196, San Miguel de Tucuman. Tucuman, Argentina. Email: [elena_sarrouf@hotmail.com](mailto:elena_sarrouf@hotmail.com)
4. MEDICUS. Larrea 877, CABA. Email: [rafael.zamora@medicus.com.ar](mailto:rafael.zamora@medicus.com.ar)
5. Sub-secretary of Primary, Ambulatory and Community care of the CABA. Monasterio 480, CABA, 1283. Email: [amirurzun@gmail.com](mailto:amirurzun@gmail.com)
6. Faculty of Medicine. University of Southampton, Southampton, UK. Email: [Nazrul.Islam@soton.ac.uk](mailto:Nazrul.Islam@soton.ac.uk)

Table of Contents

[**Supplementary Figure 1. Box-and-whisker plots of all six chosen indicators to create the HICI. City of Buenos Aires, 2020.** 2](#_Toc142401789)

[**Supplementary Figure 2: Spider chart of the relationship between indicators (Z-scores) per Comuna. City of Buenos Aires, 2020.** 3](#_Toc142401790)

# **Supplementary Figure 1. Box-and-whisker plots of all six chosen indicators to create the HICI. City of Buenos Aires, 2020.**


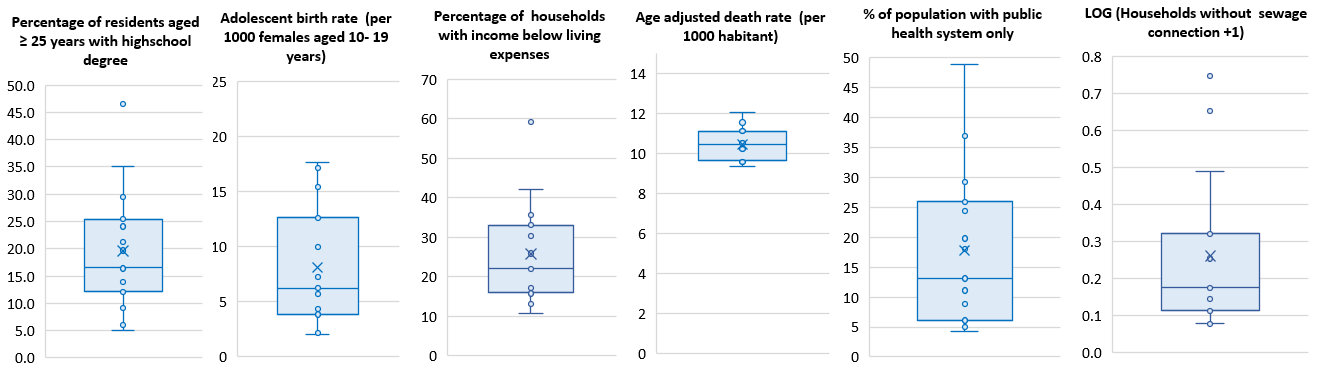


Source: generated by the authors with data available in the Direction of Statistics and Census of the City of Buenos Aires.

# **Supplementary Figure 2: Spider chart of the relationship between indicators (Z-scores) per Comuna. City of Buenos Aires, 2020.**


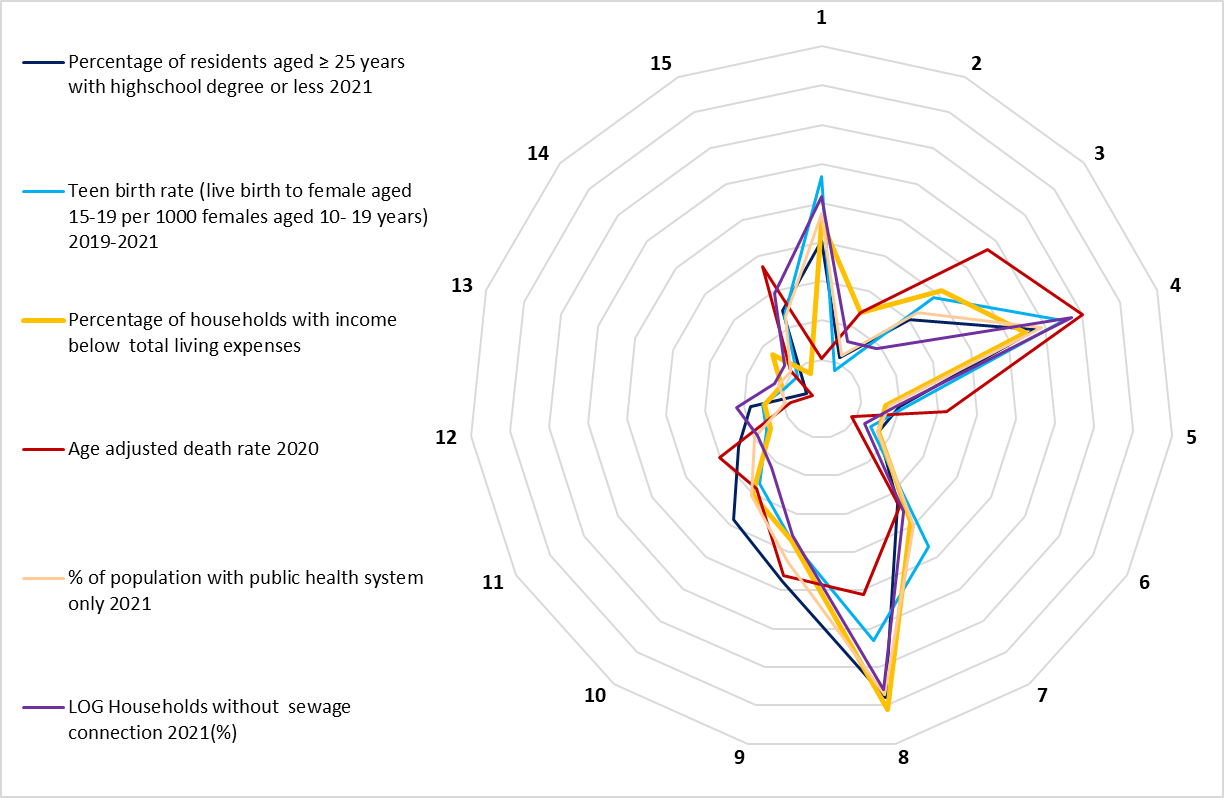


Source: generated by the authors with data available in the Direction of Statistics and Census of the City of Buenos Aires.
